# Mortality and life expectancy of Yokkaichi Asthma patients, Japan: Late effects of air pollution in 1960–70s

**DOI:** 10.1186/1476-069X-7-8

**Published:** 2008-02-26

**Authors:** Peng Guo, Kazuhito Yokoyama, Masami Suenaga, Hirotaka Kida

**Affiliations:** 1 Department of Public Health and Occupational Medicine, Mie University Graduate School of Medicine, 2-174 Edobashi, Tsu-shi, Mie 514-8507, Japan; 2 Natural Science Center for Basic Research and Development, Hiroshima University, 1-2-3 Kasumi, Minamiku, Hiroshima-shi 734-8551, Japan

## Abstract

**Background:**

The incidence of chronic obstructive pulmonary disease (COPD) and bronchial asthma began increasing in early 1960s in the population of Yokkaichi-city (Mie Prefecture, Japan). The cause of the disease was sulfur oxide air pollution, and it is known as Yokkaichi Asthma. The pollution markedly decreased by the end of 1970s; no new cases have been reported since 1988. This study aimed at examining the late effects of air pollution on the health of Yokkaichi Asthma patients.

**Methods:**

Mortality rate and life expectancy of patients, registered between 1965 and 1988, were investigated from 1975 through 2000.

**Results:**

Mortality rates for COPD and asthma in patients from Yokkaichi-city were significantly higher than in the whole population of Mie Prefecture. For all ages (except for males between 80 and 84 years in 1985), the life expectancy of both males and females were significantly reduced in patients from Yokkaichi-city as compared with the whole population of Mie Prefecture. The potential gains in life expectancy excluding the mortality for respiratory diseases including COPD and asthma were larger for all ages in patients from Yokkaichi-city.

**Conclusion:**

Mortality and life expectancy were adversely affected in patients from Yokkaichi-city, despite the fact that the air pollution problem has been already solved.

## Background

YOKKAICHI is a city with the largest population among cities and towns of Mie Prefecture, and it is located in the center of Japan. This is an industrial city and faces Ise Bay on the Pacific Ocean side of the Japanese archipelago. During World War II, naval fuel factories were constructed in the southern part of Yokkaichi Harbor, but were destroyed by bombing before they began to operate. In 1957, a petroleum complex was built and began to operate around the remnant of this facility. The complex included the largest heavy oil-fired power station and refinery in Japan at that time. As this complex used crude oil with a high sulfur content (more than 3%) and did not employ suitable measures for desulfurization, the annual sulfur dioxide (SO_2_) emission level exceeded 100,000 tons, which caused air pollution with an increased concentration of sulfur oxides, as high as 1 ppm in the polluted areas.

By early 1960s, the incidence of respiratory diseases, including bronchial asthma, increased among people living in the vicinity of the complex, and this subsequently became a major health problem in Japan, that was known as Yokkaichi Asthma(Yokkaichi Zensoku, in Japanese). A series of counter measures based on an area-wide total emission control system were taken since 1972, as a result of a successful lawsuit brought by nine inhabitants of the area against six companies in the Yokkaichi Court, for reparations of health damage caused by air pollution. Sulfur oxide air pollution in this region has been markedly reduced and the pollution reached the same level as that in un-polluted areas by the end of 1970s; atmospheric sulfur oxide level is now below 0.01 ppm in the Yokkaichi area [1]. Recently, a historical review of Yokkaichi pollution problem has been published [2].

Many investigators of Mie University [2-12] published a bulk of reports regarding health problems among residents of Yokkaichi-city up to 1990. These studies revealed that worsening of air pollution was associated with increased mortality by bronchial asthma and chronic bronchitis, and that the mortality due to bronchial asthma decreased immediately after improvement of pollution whereas the mortality by chronic bronchitis decreased to the levels of control areas 4 to 5 years after the concentration of SO_2 _was reduced to ambient air standard. Similar trends were also observed in medical consultation rates, incidence and prevalence of respiratory diseases. For example, using British Medical Research Council questionnaires, Yoshida et al [12] examined inhabitants over 40 years of age in six districts of Yokkaichi-city and reported that 5–10% of the population living in the polluted areas suffered from chronic bronchitis and obstructive diseases whereas less than 3% suffered from the same diseases in un-polluted areas. Also, Imai et al [8] observed that, during the period of 1963–82, the mortality due to chronic bronchitis was 0.82, 4.60, and 57.72 per 100,000 population between ages of 40–49, 50–59, and 60-years, respectively, in polluted areas of Yokkaichi-city, whereas it was 2.22, 1.51, and 33.82, respectively, in un-polluted areas.

To offer financial support to the patients, a Public Relief-System for air pollution was established in 1965 by the Yokkaichi-city for the first time in the world. The system was financed by the city; but in the next year, the Japanese government decided that the national treasury, Mie Prefecture and industries linked to the pollution must also finance the public relief. In 1969, this program was nationally expanded leading to the "Pollution-Related Health Damage Special Measures Law" declared by the Japanese government. Four years after, the "Pollution-Related Health Damage Compensation Law" was enacted and enforced.

The laws established that financial support to victims of environmental pollution in Japan, including Yokkaichi Asthma patients, is based on the imposition to industries, which are responsible for the pollution. Thus, medical expenses of patients from Yokkaichi-city who met the three criterions below had been paid by the program:

1) With specific diseases: Diseases (bronchial asthma, chronic bronchitis, pulmonary emphysema, and their complication) that occur excessively in the polluted areas and that have been confirmed epidemiologically.

2) In specific areas: Areas where prevalence of the specific diseases has increased.

3) During specific period: Three years of residence in the specific area.

Diagnosis of diseases was made based on clinical signs and symptoms and laboratory findings such as chest X-ray, arterial blood oxygen tension and lung function in each hospital/clinic that the patients attended. Registration and compensation were carried out since 1965; no new cases had been reported after 1988 in Yokkaichi.

It has been frequently documented that, as a traditional pollutants, both SO_2 _and suspended particulate (SP) usually occur together, representing a complex mixture produced by fossil fuel (especially coal) consumption [13-20]. Evidence has also shown that SP may exert adverse effects on health without high level of SO_2 _[16,18,20]. However, residual health effects after the improvement of pollution have not been well investigated.

The present study aimed at examining the late health effects of air pollution that occurred several decades ago in Yokkaichi. First, recent trends in mortality from respiratory diseases (i.e. chronic bronchitis, pulmonary emphysema and asthma) among registered patients were investigated. Also, life expectancy of the patients was assessed, because this is one approach to assess health effects of air pollution [21-23].

## Methods

### Subjects

Records of 1,354 patients registered in Yokkaichi-city during 1965–1988 by "Public Relief-System by Yokkaichi-city (1965)," "Pollution-Related Health Damage Special Measures Law (1969)," and "Pollution-Related Health Damage Compensation Law (1973)" were used in this study with the authorization of the Yokkaichi-city public office. Data obtained were sex, year of birth, the dates of registration and death, cause of death, and diagnosis at registration (Tables 1). Records of 1,232 patients [survival 518 (243 males and 275 females), death 714 (410 males and 304 females, on December 30, 2002)] registered after 1973 were used for the analysis and to compare with the whole population of Mie Prefecture, where the mortality data are available since 1973 [24,25].

**Table 1:** Number of death^a ^and survival^b ^among Yokkaichi Asthma patients

	Sex	1975	1980	1985	1990	1995	2000
Deaths							
Respiratory Diseases							
Chronic bronchitis	Males	4	10	6	5	2	5
	Females	1	4	8	2	8	2
Pulmonary emphysema	Males	4	2	0	3	1	2
	Females	0	0	1	3	0	0
Asthma	Males	6	15	10	20	6	6
	Females	7	9	8	10	5	7
Respiratory Cancer	Males	0	3	5	3	3	4
	Females	0	1	0	2	0	1
Others^c^	Males	5	9	18	12	11	6
	Females	7	1	11	5	4	7
Other than respiratory	Males	44	52	39	39	27	23
diseases	Females	26	26	32	36	32	38
Total	Males	63	91	82	82	50	46
	Females	41	41	58	58	49	55

Survivals	Males	396	439	389	389	327	281
	Females	369	417	422	422	372	317

^a^Sum over 5 years (± 2 years of each year)

^b^On October 1.

^c^Respiratory diseases other than the above-mentioned disorders.

The population of Yokkaichi-city was 218,981(male 105,468; female 113,513), 247,001 (male 120,893; female 126,108), 263,001(male 127,876; female 135,125) and 274,180 (male 134,161; female 140,019), in 1965, 1975, 1985 and 1990, respectively. The population of Mie Prefecture was 1,514,467 (male 727,802; female 786,665), 1,626,002 (male 787,280; female 838,722), 1,747,311 (male 847,420; female 899,891) and 179,2514 (male 869,515; female 922,999), in 1965, 1975, 1985 and 1990, respectively [25].

### Age-adjusted mortality rate (indirect method)

Age-adjusted mortality rates of patients for all and specific causes were calculated by the indirect method [26] using age-specific mortality rates of the whole population of Mie prefecture (standard population) in the census years of 1975–2000 (every 5th year). In this calculation, the number of patients in each age-group was the average over ± 2 years of the census year. Chronic bronchitis and pulmonary emphysema were included in the group with chronic obstructive pulmonary disease (COPD) by ICD10 [27], and the comparison between patients from Yokkaichi and the whole Mie Prefecture was performed in groups with COPD and asthma, respectively.

### Calculation of life expectancy by life-table method

The abridged life table method [28-31] was used to calculate the life expectancy of patients with ages of 0–84 years (5-year intervals). The fraction of the last age interval of life [28] was used to construct an abridged life table. Those fractions were calculated from a complete life table in the Japan census years of 1975–2000 (every 5th year) [32]. The number of patients was calculated on October 1 of each year; the number of death was the average over ± 2 years of the census year. Standard error (SE) for life expectancy was estimated by Chiang method [28-31,33,34]. By the same method, the life expectancy of the whole population of Mie prefecture was calculated based on data of Mie Prefecture [25] registered in the same period.

### Potential gains in life expectancy

Techniques for partial multiple decremental life tables [29,35] were used to calculate the corresponding life expectancies of patients from Yokkaichi and from the whole population of Mie Prefecture by excluding the death due to respiratory diseases such as chronic bronchitis, pulmonary emphysema, asthma, pneumonia and acute bronchitis. The gain in life expectancy was calculated as the difference between the life expectancies with and without these causes of death.

## Results

**Figure 1:**
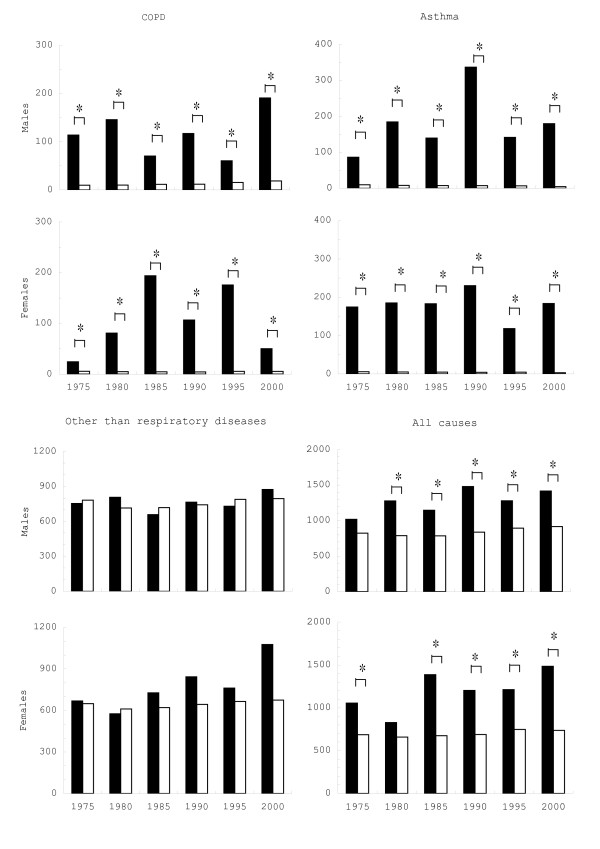
Age-adjusted mortality rates in Yokkaichi Asthma patients and in the whole population of Mie Prefecture. Per 100,000 population. Closed = patients, open = Mie Prefecture. COPD = chronic bronchitis and pulmonary emphysema. *p < 0.05.

For all causes, mortality rates were significantly higher in patients from Yokkaichi than in the general population of Mie Prefecture, excepting males in 1975 and females in 1980 (Fig 1). Mortality rates for COPD and asthma in patients from Yokkaichi (116.29 and 105.11 per 100,000 in average for males and females, respectively) were approximately 10- to 20-fold higher than in the general population of Mie Prefecture (12.95 and 4.92 per 100,000 in average for males and females, respectively). Similarly, the mortality for asthma in the Yokkaichi patients (178.0 and 179.0 in average for males and females, respectively) was more than 20-folds higher than in the general population of Mie Prefecture (7.26 and 4.46 in average for males and females, respectively). By contrast, the mortality rates for all other causes were not significantly different between the two groups.

**Figure 2:**
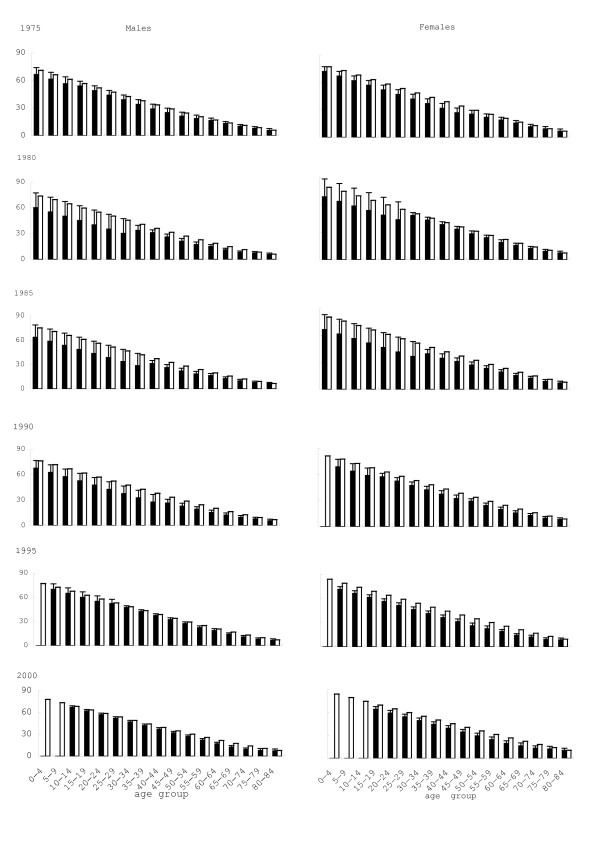
Life expectancy for Yokkaichi Asthma patients and whole population of Mie Prefecture. Average with standard error of mean (SEM). SEM was less than 0.04–0.19 years for Mie Prefecture. Closed = patients, open = Mie Prefecture. Significantly different (p < 0.05) between Yokkaichi patients and Mie population for all age subgroups except for males aged 80–84 years in 1985 (t-test).

**Figure 3:**
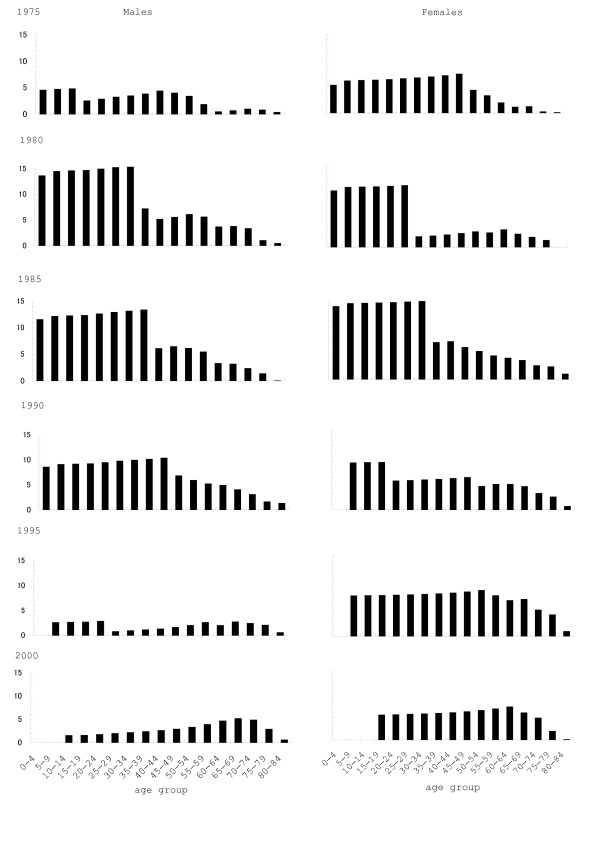
Differences in life expectancy (years) between Yokkaichi patients and Mie Prefecture. Blanks = unable to calculate because of no deaths.

The life expectancy for Yokkaichi patients and Mie Prefecture is shown in Fig 2. For all ages (except for males of 80–84 years in 1985), the life expectancy of both males and females was significantly reduced in Yokkaichi patients as compared with the whole population of Mie Prefecture, for 1975–2000. The average difference in males of each age group was 2.72, 8.50, 7.88, 6.95, 1.92 and 2.80 years in 1975–2000, respectively; and in females, the difference was 4.52, 5.27, 8.54, 5.31, 6.81 and 5.26 years, respectively. As shown in Fig 3, difference in life expectancy was larger in younger patients; differences among age-groups became smaller during the observation period. The whole life table is given as an Appendix (see Additional file 1).

The potential gain in life expectancy for Yokkaichi patients and for the population of Mie Prefecture after excluding mortality for respiratory diseases (see above) is shown in Table 2. Yokkaichi patients showed greater gains as compared with those from Mie Prefecture, i.e. 2.33–3.32 and 1.51–4.19 years on average, for males and females, respectively. The gain was less than 0.65 year for the general population of Mie Prefecture in all age groups.

**Table 2:** Potential gains in life expectancy(years) after excluding mortality for respiratory diseases^a^

	1975		1980		1985		1990		1995		2000	
Ages(years)	Patients	Mie	Patients	Mie	Patients	Mie	Patients	Mie	Patients	Mie	Patients	Mie

Males												
0–4	-	0.50	-	0.50	-	0.52	-	0.59	-	0.64	-	0.62
5–9	-	0.49	-	0.45	-	0.51	-	0.57	-	0.63	-	0.60
10–14	-	0.48	-	0.45	-	0.51	-	0.57	-	0.63	-	0.60
15–19	-	0.46	-	0.44	-	0.50	-	0.57	-	0.63	-	0.60
20–24	-	0.46	-	0.44	-	0.50	-	0.57	3.54	0.62	-	0.60
25–29	-	0.46	-	0.44	-	0.49	-	0.56	-	0.62	-	0.60
30–34	-	0.46	2.89	0.44	-	0.49	-	0.55	-	0.61	-	0.60
35–39	-	0.45	3.77	0.44	-	0.48	-	0.55	-	0.60	-	0.59
40–44	3.18	0.45	-	0.44	-	0.48	-	0.55	-	0.60	-	0.59
45–49	3.30	0.45	-	0.43	4.30	0.48	-	0.55	-	0.60	-	0.59
50–54	3.47	0.45	4.05	0.43	4.46	0.48	5.19	0.55	-	0.59	-	0.60
55–59	3.94	0.45	4.31	0.43	4.78	0.48	4.68	0.55	3.70	0.59	-	0.59
60–64	1.94	0.44	3.85	0.43	4.50	0.47	4.50	0.54	3.13	0.59	-	0.59
65–69	2.17	0.41	3.23	0.41	3.82	0.47	3.38	0.53	3.21	0.57	2.99	0.57
70–74	1.45	0.36	2.83	0.37	2.67	0.43	3.23	0.49	3.22	0.52	2.73	0.54
75–79	1.08	0.25	1.72	0.29	1.46	0.34	1.39	0.41	2.53	0.42	2.84	0.44
80–84	0.46	0.11	0.77	0.13	0.60	0.16	0.89	0.19	0.93	0.21	0.91	0.23
Average	2.33	0.42	3.05	0.41	3.32	0.46	3.32	0.52	2.90	0.57	2.37	0.56

Females												
0–4	-	0.36	-	0.33	-	0.32	-	0.34	-	0.34	-	0.32
5–9	-	0.32	-	0.30	-	0.31	-	0.32	-	0.34	-	0.31
10–14	-	0.31	-	0.30	-	0.30	-	0.32	-	0.34	-	0.31
15–19	-	0.31	-	0.30	-	0.30	-	0.32	-	0.34	-	0.31
20–24	-	0.31	-	0.30	-	0.30	-	0.31	-	0.34	-	0.31
25–29	-	0.30	2.20	0.29	-	0.29	-	0.30	-	0.34	-	0.31
30–34	-	0.30	-	0.29	13.52	0.29	-	0.30	-	0.33	-	0.31
35–39	-	0.30	-	0.29	-	0.29	-	0.30	-	0.33	-	0.30
40–44	-	0.30	-	0.28	5.53	0.29	-	0.29	-	0.33	-	0.30
45–49	4.57	0.29	-	0.28	-	0.28	2.92	0.29	-	0.32	-	0.30
50–54	4.14	0.28	2.72	0.28	3.14	0.28	-	0.28	3.25	0.32	-	0.30
55–59	3.48	0.27	-	0.28	3.30	0.28	2.03	0.28	1.94	0.31	-	0.29
60–64	3.85	0.26	2.79	0.27	3.45	0.27	2.08	0.27	2.07	0.31	2.22	0.28
65–69	2.67	0.25	2.12	0.25	3.22	0.25	2.21	0.26	2.12	0.30	2.46	0.27
70–74	2.45	0.22	1.74	0.23	2.29	0.23	1.82	0.23	2.18	0.27	1.43	0.25
75–79	1.97	0.17	1.74	0.17	2.09	0.18	1.86	0.19	2.12	0.22	1.12	0.20
80–84	0.97	0.09	0.70	0.09	1.16	0.09	0.94	0.09	1.03	0.11	0.30	0.11
Average	3.01	0.27	2.00	0.27	4.19	0.27	1.98	0.28	2.10	0.30	1.51	0.28

^a^See Methods section.

- = Unable to be calculated because of no deaths.

## Discussion

The mortality rate for COPD and asthma in Yokkaichi patients was significantly higher than in the whole population of Mie Prefecture for 1975–2000. However, no significant differences were observed among other illnesses. Yokkaichi patients also showed shorter life expectancy than the whole population of Mie Prefecture. As life expectancy was calculated based on the death rate, it appears that shortening of life expectancy reflects high mortality rates in patients from Yokkaichi.

This observation agrees with the fact that potential gain in life expectancy is more greatly prolonged in patients from Yokkaichi than in the general population of Mie Prefecture when death for respiratory diseases was excluded in both males and females. The excess in gains of Yokkaichi patients reached approximately half of the difference in life expectancy between Yokkaichi patients and Mie Prefecture (Table 2 and Fig 3), suggesting that decrease in life expectancy was greatly due to respiratory mortality. Thus, the present study revealed that delayed adverse health effects still persisted among victims despite reduction in air pollution and no further incidence of COPD and asthma since 1988.

Several groups reported reduced life expectancy among people living in air-polluted areas. Decrease of 1.51 year was reported in Dutch men [21], 1.34–1.69 years in Shanghai [22], and 2.5–3.1 years in US population [36]. Patients from Kurashiki-city, Japan, that suffered from respiratory diseases due to air pollution, have reported life expectancy of 5.1 and 7.3 years (males and females) shorter than the general Japanese population [23]. Loss of life expectancy has been also reported in smokers. i.e. 3.5 and 2.2 years in 40 years old men and women in the Japanese [34] and 8.6 years in the US population [36]. The changes in life expectancy among subjects aged 40 or less found in the present study seem much greater than these observations, up to 1990. If the pollution in Yokkaichi-city was not reduced by the end of 1970s, more magnitude of adverse health effects could have been observed among the victims. Although it has been argued that COPD models lack good data on life expectancy [37], estimation of life expectancy and its potential gains after excluding specific causes appear to be useful for assessing the effects of air pollution on health.

The studies on Yokkaichi Asthma had focused mainly on the effects of SO_2_and/or sulfuric acid mists [2,8,38] whereas those in other countries have shown that SO_2 _+ SP or SP alone lead to adverse health effects [13-20]. However, monthly prevalence of respiratory disease during the period from 1962–65 was significantly correlated with the corresponding values of SO_2_as well as with the amount of dust fall in the Yokkaichi area, although the level of total SP and dust fall in this area were reported to be low [3,8]. It had been suspected that low pH value of dust contributed to Yokkaichi Asthma problems [8,38].

It has been demonstrated that SO_2 _causes increased cardiovascular mortality [17,19,39] and hospital admission rate [19,40,41]. By contrast, no increased cardiovascular mortality has been observed in residents of Yokkaichi[11] where elevated mortality for COPD and asthma has been reported [2-12]. Increase in cardiovascular mortality has not been demonstrated in the registered patients of Yokkaichi Asthma [4,9,10]. The reason for this discrepancy remains to be elucidated.

In the present study, there could have had an error in the estimates of life expectancies by the Chiang method when the population size is smaller than 1000 [42]. Because of the small size of subjects, the statistical analysis should have been done by Kaplan-Meier method for comparing the survival curves between the patients and healthy people during the same period, but these are not available.

The most important risk factor of COPD in the developed world is cigarette smoking [43,44]. Other risk factors are occupational or environmental exposure to dust, gas, vapor or fumes [45], malnutrition [46] and increased airway responsiveness [47]. Further, asthmatics are sensitive to air pollutants such as SO_2_, SP, ozone and nitrogen dioxide [48]. The effects of these risk factors on the patients should be evaluated in future studies. Since prognosis of COPD can be predicted by lung function such as FEV_1 _[49,50], analysis using lung function should be carried out in Yokkaichi Asthma patients. Adequate measures for health protection are also important to investigate.

## Conclusion

Mortality and life expectancy were adversely affected in the Yokkaichi Asthma patients due to increase in death for respiratory diseases despite the fact that the problem of air pollution has been already solved.

## Competing interests

The author(s) declare that they have no competing interests.

## Authors' contributions

PG carried out statistical analyses including life-table methods and prepared the first draft. KY was the mentor of the study, participated in its design and coordination, and helped in the preparation of the manuscript. MS wrote a program for the life-table analysis. HK participated in data collection and coordination of the study. All authors read and approved the final manuscript.

## Supplementary Material

Additional file 1 Appendix. The data provided whole life table for Yokkaichi Asthma patients and population of Mie Prefecture. Click here for file
